# Bifunctional 1,2,4-Triazole/12-Tungstophosphoric Acid Composite Nanoparticles for Biodiesel Production

**DOI:** 10.3390/nano12224022

**Published:** 2022-11-16

**Authors:** Gicheon Lee, Chanmin Lee, Hyungjin Kim, Yukwon Jeon, Yong-Gun Shul, Jinwon Park

**Affiliations:** 1 Department of Chemical and Biomolecular Engineering, Yonsei University, Yonsei-ro 50, Seodaemun-gu, Seoul 120-749, Republic of Korea; 2 Green and Sustainable Materials R&D Department, Korea Institute of Industrial Technology, 89 Yangdaegiro-gil, Ipjan-myeon, Seobuk-gu, Cheonan-si 31056, Chungcheongnam-do, Republic of Korea; 3 Department of Environmental and Energy Engineering, Yonsei University, 1 Yonseidae-gil, Wonju 26493, Republic of Korea

**Keywords:** acid–base bifunctional composite, heteropoly acid, triazole, biodiesel, transesterification

## Abstract

Here, a composite nanoparticle with an acid–base bifunctional structure has been reported for the transesterification of rapeseed oil to produce biodiesel. Triazole-PWA (PWA = 12-tungstophosphoric acid) composite materials with a hexahedral structure are produced using the precipitation method, showing the average particle diameters of 200–800 nm. XPS and FT-IR analyses indicate well-defined chemical bonding of triazole moieties to the PWA. The functionalization and immobilization of PWAs are investigated due to strong interactions with triazole, which significantly improves the thermal stability and even surface area of the heteropoly acid. Furthermore, various ratios of triazole and PWAs are examined using NH_3_-TPD and CO_2_-TPD to optimize the bi-functionality of acidity and basicity. The prepared nanomaterials are evaluated during the transesterification of rapeseed oil with methanol to analyze the effect of triazole addition to PWAs according to the different ratios. Overall, the bifunctional triazole-PWA composite nanoparticles exhibit higher fatty acid methyl ester (FAME) conversions than pure PWA nanoparticles. The optimized catalyst with a triazole:PWA ratio of 6:1 exhibits the best FAME-conversion performance due to its relatively large surface area, balance of acidity, and strong basicity from the well-designed chemical nano-structure.

## 1. Introduction

Composites, composed of two or more chemically distinct components that possess properties not available from the components acting individually, have been steadily researched in medicine, bio, energy, polymers, etc., with unlimited potential for development [1,2,3,4,5,6]. In the last decade, inorganic–organic composite nanomaterials have been extensively used for fine chemical production, such as biodiesel, because of their molecular hybrid networks, physical and catalytic properties, and material selectivity [7,8,9,10,11,12]. Biodiesel production has gained prominence due to an increase in the demand and cost of energy, environmental considerations, and the depletion of fossil-fuel reserves. Methyl esters of long-chain fatty acids, known as biodiesel, are synthesized by the catalytic transesterification reactions of renewable triglycerides and methanol in the presence of acid or alkaline catalysts [13]. Generally, transesterification occurs in three steps: triglycerides are sequentially converted to diglycerides, monoglycerides, and glycerol (a by-product), with alkyl ester (biodiesel) production at every step [14]. Biodiesel production involves the reaction of lipids with an alcohol in the presence of an acidic or alkaline catalyst [15]; thus, the catalysts for biodiesel production (classified as acid, base, or enzyme catalysts) have attracted immense attention. As basic catalysts (such as sodium and potassium hydroxide, methoxide, and carbonate) cause high reaction rates, they are generally used for industrial biodiesel synthesis [16]. However, very little water causes basic catalysts to undergo the saponification reaction, making anhydrous conditions essential for basic catalysis [17]. Contrarily, esterification and transesterification occur simultaneously in acid-catalyzed biodiesel production. Furthermore, the process enables transesterification in the presence of water [18].

Currently, homogeneous catalysts are commonly used for industrial synthesis because of their relatively short reaction times (<1 h) [19]. However, they are associated with several limitations, such as corrosion, environmental pollution due to alkaline and acidic wastewater, and difficult separation of the catalyst from the product [20,21]. Therefore, solid-catalyst systems have been proposed for efficient biodiesel production. Heterogeneous solid catalysts exhibit several advantageous properties compared to homogenous systems, such as the production of high-quality ester products, glycerol by-products, and a relatively facile separation from the product. Therefore, to ensure high catalytic-reaction efficiency, the activity and selectivity of the catalyst, which are largely dependent on the catalytic-material design (with a dispersion of the active sites), should be improved.

Heteropoly acids (HPAs), with the Keggin structure, exhibit high catalytic activity owing to their strong acidity [22]. However, HPAs exhibit several limitations, such as low thermal stability, low surface area, and high solubility [23]. To overcome these limitations, HPAs have been modified with various supports, such as zeolite and carbon; modified HPAs, with the hydrogen ions substituted by metals, organic materials, and ionic liquids, exhibit a large surface area [24,25,26,27,28]. Li et al. [29] have synthesized Zn_1.2_H_0.6_PW_12_O_40_ nanotubes using cellulose-fiber templates; they exhibit double acid sites (Lewis acid and Brønsted acid sites) with a higher catalytic activity than H_3_ PW_12_O_40_ in the conversion of waste cooking oil. Additionally, a recently reported acid–base bifunctional HPA (which combines rare-earth metals into a heteropoly structure) exhibits enhanced catalytic activity for one-pot tandem reactions due to its tunable antagonistic functions [30]. However, further research is required on modulating these HPA-based chemical antagonistic functions.

In this study, a nanocomposite of triazole-incorporated heteropoly acid was synthesized by combining 1,2,4-triazole (triazole) with 12-tungstophosphoric acid (PWA) using the precipitation method for the transesterification of rapeseed oil. Physical and chemical characterizations using different triazole/PWA ratios were used to optimize the triazole-PWA bifunctionality. Furthermore, to investigate the acid–base composites as catalysts for biodiesel production, the transesterification of rapeseed oil in the presence of the triazole/PWA particles was analyzed under various reaction conditions. Heteropoly acid-immobilized solid nanoparticles exhibited high catalytic activity due to their large surface areas and high chemical and thermal stability.

## 2. Materials and Methods

### 2.1. Synthesis of the Heterocycle–Heteropoly Acid

12-Tungstophosphoric acid (98%, Kanto Chemical, Tokyo, Japan) and 1,2,4-triazole (98%, Sigma-Aldrich, St. Louis, MO, USA) were used in this study. A PWA solution (0.03 M) was added into a 1,2,4-triazole solution (0.03 M), stirred for 6 h, centrifuged at 3000 rpm to separate impurities, and dried at 100 °C overnight. A subsequent heat treatment at 400 °C for 3 h produced the triazole/PWA nanocomposite. We refer to the triazole-incorporated heteropoly acid nanocomposite as PWT corresponding to triazole amounts in synthesis. The PWTx indicates the triazole-incorporated PWA nanoparticles prepared with specific heteropoly acid amounts (PWT1 is from a triazole:PWA ratio of 1:1, PWT2 is from a triazole:PWA ratio of 3:1, PWT3 is from a triazole:PWA ratio of 4:1, PWT4 is from a triazole:PWA ratio of 6:1, and PWT5 is from a triazole:PWA ratio of 10:1).

### 2.2. Characterization

The surface areas of the synthesized catalysts were evaluated by N_2_-adsorption measurements at liquid-nitrogen temperature. The Brunauer–Emmett–Teller (BET) surface areas of PWTx were in the range of 3.99–8.18 m^2^/g. X-ray diffraction (XRD) patterns were collected at room temperature using a Japanese Rigaku Miniflex using CuKa radiation. IR spectra (500–4000 cm^−1^) were recorded using KBr discs by a Nicolet Magna 560 IR spectrometer. A Shimadzu DT 50 thermal analyzer was used for thermogravimetric analysis (TGA) and differential thermal analysis (DTA) in air condition. Scanning electron micrographs (SEM) were recorded using a JEOL-6701F microscope. Element analysis was performed on a 2400 Series II from PerkinElmer. The pH values of the solution after synthesis were determined using a pH meter (Orion Star A211, Thermo Fisher, Waltham, MA, USA). X-ray photoelectron spectroscopy (XPS) was measured using a K-alpha XPS spectrometer (Thermo Fisher) equipped with a mono-chromatic Al Kα X-ray source. XPS peaks were fitted by the Fityk program using Shirley background subtraction and a Gaussian function.

Acidic–basic properties were analyzed by NH_3_ and CO_2_ adsorptions, respectively, using a Bel-Cat-M (BEL JAPAN INC., Toyonaka-shi, Japan) instrument loaded with 50 mg of the sample at 80 °C. In each case, the sample was treated at 100 °C for 60 min under He (50 mL); flushed with NH_3_ and CO_2_, respectively, at 80 °C for 60 min; and cooled to room temperature. Subsequently, the system was flushed with He, and the temperature was increased to 850 °C at a rate of 5 °C/min and maintained for 10 min. The effluent gases were continuously analyzed for NH_3_ and CO_2_, respectively, using a thermal-conductivity detector with the aforementioned method used for NH_3_ and CO_2_ adsorptions to analyze the acidic–basic properties. Blank tests were performed at the same conditions with only He.

### 2.3. Transesterification of Rapeseed Oil

The catalytic activity of the modified heteropoly acid was investigated by the transesterification of commercial rapeseed oil purchased from the market. Rapeseed oil (10 g) and the catalyst (0.4 g) (4 wt%) were loaded into a batch reactor (HR-8200, Hanwoul, Gunpo-si, Korea) and stirred vigorously. After the catalyst was evenly mixed, specific amounts of methanol were added to the reactor. The reaction was initiated by heating the reactor under vigorous stirring to a certain temperature (in the range of 80–240 °C) for 2 h. After transesterification, the products were separated from the catalyst by filtration, and the esters in the products were extracted by the addition of dichloromethane (as the organic-phase solvent). Dichloromethane was removed from the organic layer by heating it to 70 °C.

The fatty acid methyl ester (FAME) content of the organic layer was determined following the European-regulated procedure, EN 14103. The organic layer (250 mg) was added to 5 mL of a heptane (C7) solution of the internal standard, methyl heptadecanoate (10 g/L of the C17 ester in heptane). The FAME content (wt%) was calculated using the following formula:

(1) $$\mathrm{FAME}\;\mathrm{Content}\;\left(\mathrm{wt}\%\right)=\frac{\left(\sum A_{i}-A_{Mh}\right)}{A_{Mh}}\times \frac{C_{Mh}\times V_{Mh}}{m}\times 100$$

Here, $\sum A_{i}$ is the total peak area from methyl ester, from methyl miristate (C14) to methyl nervonate (C24:1); *A_Mh_* is the area of methyl heptadecanoate, whose response factor is equal to that of the FAME; *C_Mh_* is the concentration in mg/mL of methyl heptadecanoate (10 mg/mL); *V_Mh_* is the volume (in mL) of the methyl heptadecanoate solution (5 mL); and m is the weight (mg) of the sample (250 mg). This solution was analyzed using an Acme 6000 GC with a GL Science InertCap^®^ WAX capillary column (GL Science, Tokyo, Japan).

## 3. Results and Discussion

### 3.1. Synthesis and Characterization of the PWTx Bifunctional Catalysts

In this study, an acid–base bifunctional nanocomposite was used to overcome the drawbacks of PWAs, such as their high solubility, low surface area, and low chemical stability. PWTx bifunctional solid nanoparticles were synthesized using the precipitation method [31], as described in Section 2.1. To optimize the triazole-PWA composites, PWTx nanomaterials with different stoichiometric ratios (Triazole/PWA = 1, 3, 4, 6, and 10) were synthesized. In Table 1, it can be seen by the elemental analysis (EA) that the mass ratio of carbon and nitrogen in the composite is kept constant regardless of the amount of triazole added. Theoretically, when the PWA containing three protons is substituted with triazole, it should have a mass ratio of 2.26% and 3.95% for carbon and nitrogen, respectively, meaning that the excess triazole is dissolved in the solution without binding to the PWA. This can be proved by the pH change from 1 to 4 and 5, as in Table 1.

**Figure 1:**
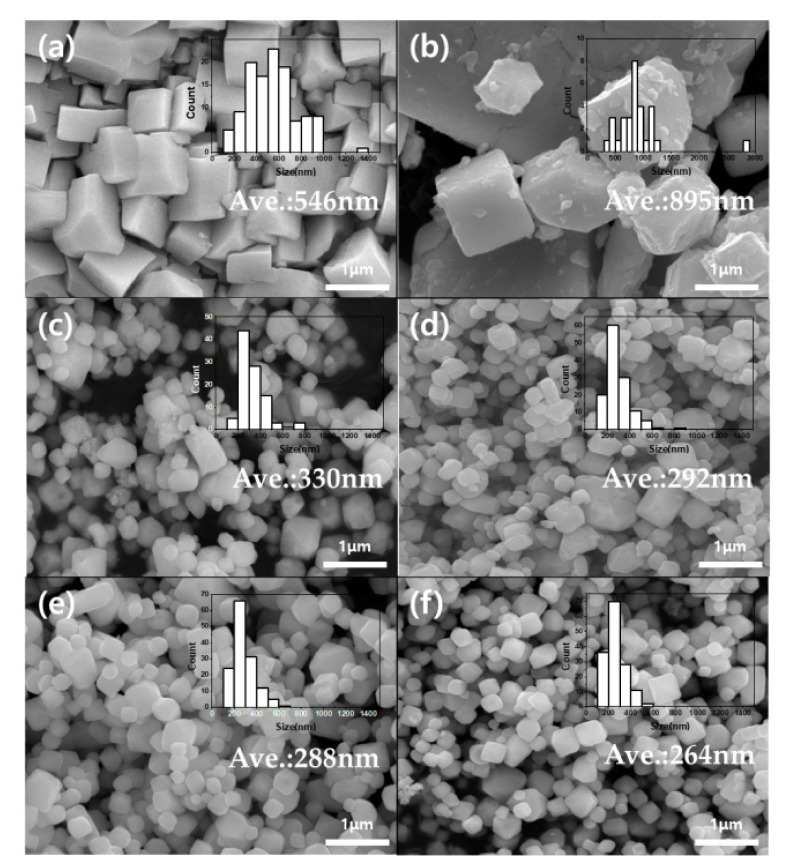
SEM images of (**a**) PWA, (**b**) PWT1, (**c**) PWT2, (**d**) PWT3, (**e**) PWT4, and (**f**) PWT5.

Figure 1 shows the morphology of the PWTx composites, as indicated by field-emission scanning electron microscopy (FE-SEM). The pure PWA exhibited an average particle diameter of ~546 nm (Figure 1a), while the triazole-modified PWA samples exhibited obvious hexahedral structures with a reduction in the average particle diameter from 895 nm (PWT1) to 330 nm (PWT2), 292 nm PWT3, 288 nm (PWT4), and 264 nm (PWT5) when increasing the number of triazole moieties. Thus, the decrease in the particle size as the specific gravity of triazole increased is due to pH changes in synthesis conditions. In general, a PWA with three protons can interact with up to three triazoles. A.A. Baranov et al. reported that the pH of the solution could affect the substitution reaction of a PWA as a result of a sharp decrease in average size from the maximum (1325 nm) to the minimum (170 nm) while the pH increased from 0 to 2 [32]. As in Table 1, the excess triazoles in the solution affect the pH increases of the solution where no PWAs are left to react. Therefore, it can be inferred that this increase in pH by dosing triazole may result in a decrease in particle size.

**Figure 2:**
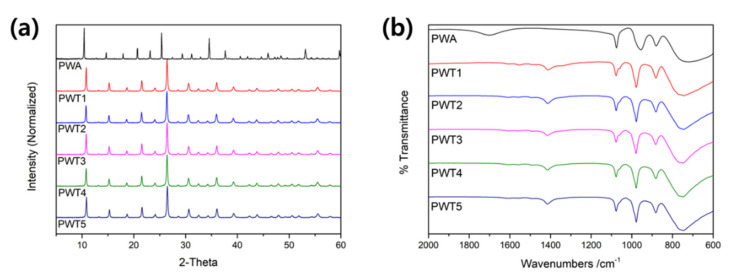
(**a**) X-ray diffraction and (**b**) FT-IR spectra of the PWTx samples.

For structural characterization, the XRD spectra of PWA and the PWTx samples were compared. As shown in Figure 2a, the tungstophosphoric acid showed diffraction peaks at 10.41, 14.68, 17.92, 20.70, 23.16, 25.37, 27.44, 29.34, 31.17, 32.89, and 34.56° (JCPDS no. 75-2125), while the triazole-incorporated PWA showed crystalline peaks at different positions; PWT1 exhibited diffraction peaks at 10.80, 15.22, 18.65, 21.53, 24.11, 26.42, 28.21, 30.62, 32.00, 33.81, and 36.00°, respectively. These findings indicated that all the synthetic materials maintained the primary structure of the HPA, even after forming an incorporated structure. Additionally, the main peaks in the sample (with different compositions) spectra exhibited a right shift compared to those in the pristine-PWA spectrum; this could be attributed to a change in the cell parameter, confirming the interaction of triazole with the heteropoly acid anion. Figure 2b shows the FT-IR spectra of the synthesized composites (with different molar ratios of PWA and triazole). The pure PWA exhibited four characteristic peaks at 1076, 956, 881, and 723 cm^−1^, corresponding to the four vibrations of oxygen atoms in PW_12_O_40_^3−^, which could be attributed to the asymmetric vibrations of P-O_a_ (the internal oxygen connecting P and W), W-O_d_ (the terminal oxygen bonding to the W_atom_), W-O_b_ (the edge-sharing oxygen connected to W), and W-O_c_ (the corner-sharing oxygen connecting the W_3_O_13_ units) [33]. Thus, the five composites with different ratios maintained the Keggin structure of the PWA, thereby exhibiting its four characteristic peaks. Additionally, the 956 cm^−1^ band of the pure PWA shifted to 978 cm^−1^ in all the composites, indicating a change in the binding energy of the terminal O-W bond; this could be due to interactions between the W atoms in the PW_12_O_40_^3−^ anion and triazole atoms. Additionally, it must be noted that the XRD and FT-IR results show almost no difference between the samples with different amounts of triazole, confirming that the optimized chemical bonding structure of the PWA with three protons interacted with triazole during the precipitation method.

**Figure 3:**
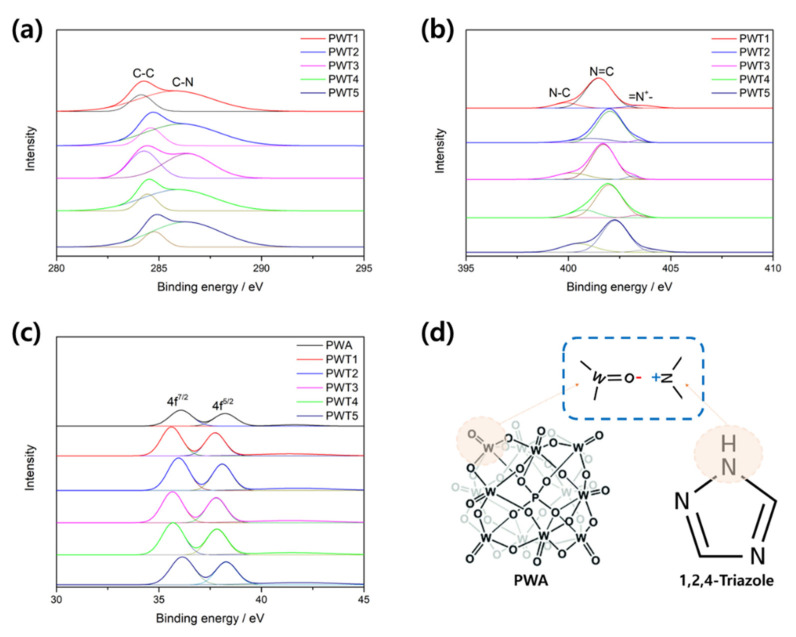
XPS spectra of PWA and the as-synthesized catalysts: (**a**) C 1s, (**b**) N 1s, and (**c**) W 4f. (**d**) Illustration of the plausible chemical bonding in PWTx.

The chemical states of the PWA and triazole-functionalized PWA were verified by X-ray photoelectron spectroscopy (XPS). As shown in Figure 3a for the C 1s spectrum, two peaks associated with C–C and C–N were observed at approximately 284–284.7 and 285.8–286.3 eV, respectively [34]. This change in the C-species bonding indicated the presence of a triazole ring in the triazole-PWA composites. As shown in Figure 3b for the N 1s spectrum, three peaks at approximately 400–401, 401.4–402.2, and 403.1–403.9 eV were observed for the triazole-PWA composites and attributed to C-N, -N= (imine nitrogen) and -N^+^- (positively charged nitrogen), respectively. After triazole functionalization, the XPS spectrum of N 1s shifted to higher binding energies (by approximately 2 eV), indicating the charge transfer of a strong π-π conjugate interaction between the nitrogen atom in triazole and the terminal oxygen of the PWA [35].

Contrarily, the binding energies of W 4f in Figure 3c were shifted to lower values in the triazole-PWA composites; this could be due to interactions between the Keggin PWA and carbon framework, enhancing the catalyst stability [36]. FT-IR and XPS analyses indicated that the triazole and PWA-synthesized composites maintained a PWA-specific structure. Furthermore, the terminal oxygen of the PWA electrostatically reacted with the non-bonding nitrogen of triazole, as shown in Figure 3d.

**Figure 4:**
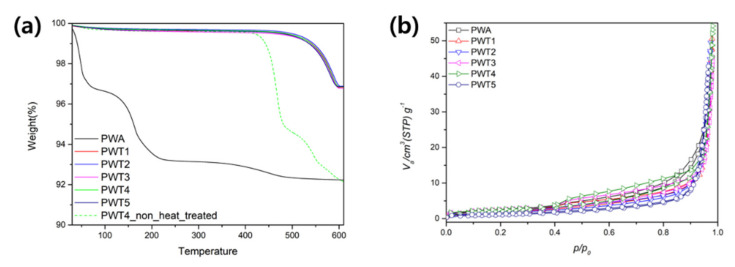
(**a**) Thermogravimetric variations exhibited by the PWTx composites and pristine PWA under air. (**b**) Nitrogen gas adsorption–desorption isotherms of the PWA and the synthesized catalysts.

A thermogravimetric analysis (TGA) of the PWA and the triazole-PWA composites was used to analyze their thermal stability. As shown in Figure 4a, the TGA profile of the pristine PWA showed three steps of weight change. A weight loss was observed at ~9 wt%; it occurred by a two-step process below 250 °C and could be attributed to the water of crystallization. Additionally, a gradual weight loss in the temperature range of 300–500 °C could be attributed to the elimination of constitutional water [37]. Compared to the pure PWA, the TGA curves of triazole-modified PWA exhibited two main weight losses at approximately 450 and 600 °C. Thus, the triazole functionalization of the PWA particles significantly improved their thermostability, despite low boiling point of triazole (260 °C).

The surface and pore-structure properties of the pure PWA and triazole-modified PWA were analyzed using BET surface area, pore volume, and pore diameter values determined using nitrogen adsorption–desorption isotherms (Figure 4b and Table 2). The nitrogen sorption curves for the pure PWA, PWT1, PWT2, PWT3, and PWT4 exhibited type-III isotherms and H3 hysteresis loops, corresponding to a microporous structure [38]. In contrast, a reduction was observed in the nitrogen sorption isotherm of PWT5; this could be attributed to the destruction or obstruction of the porous structure due to the excessive addition of triazole. Additionally, the surface area, pore volume, and pore diameter values exhibited similar overall trends, as summarized in Table 1. The surface area and pore volume values increased from 6.8811 m^2^/g and 1.581 cm^3^/g for PWT1 to 8.181 m^2^/g and 1.8796 cm^3^/g for PWT4, respectively, whereas PWT4 A and PWT5 exhibited lower values of 3.9909 m^2^/g and 0.9169 cm^3^/g, respectively, possibly due to the over-supply of triazole.

**Figure 5:**
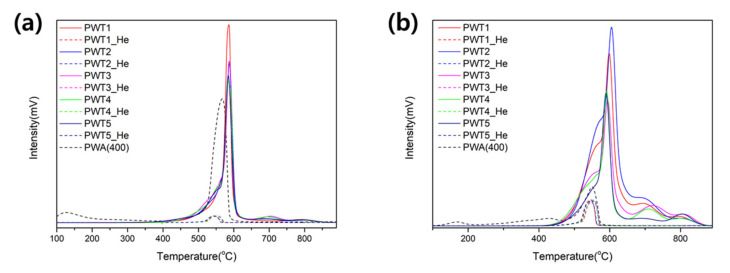
The temperature-programmed desorption of PWTx using (**a**) NH_3_ and (**b**) CO_2_.

Figure 5 shows the NH_3_ and CO_2_ temperature-programmed desorption (TPD) profiles. The peak areas and positions indicate the catalyst acidity, which are important parameters indicating the catalytic activity [39]. The number of basicity and acidity sites was calculated by integrating the desorption area; Table 3 lists the total acidity and basicity capacities.

To verify the acidic properties of PWTx, the NH_3_-TPD profiles were analyzed by increasing the temperature. The dashed lines included in the two graphs (Figure 5) are the temperature-dependent behavior of each sample without adsorbing any gas, indicating almost no decomposition of the developed composite structure. As shown in Figure 5a, all the curves exhibited a main peak at around 580 °C, with PWT1 exhibiting the highest peak intensity. However, as seen in the calculated values in Table 3, all the samples revealed similar acidity, regardless of the triazole ratio. In addition, when compared with the peak behavior of the heat-treated PWA under the same conditions, it can be confirmed that the acidic capacity was maintained, although the peak position shifted. Thus, the Brønsted acid sites of the PWA remained constant in spite of the triazole content of PWTx.

Figure 5b shows the CO_2_-TPD profiles to investigate the basic properties of PWTx. Although the desorption intensities are in order of PWT2, PWT1, PWT3, PWT4, and PWT5, the ratio of acid sites to base sites is estimated and shows a constant ratio of 0.4–0.7 by comparing the basicity calculated from the desorption areas (Table 3). Interestingly, the proportion of strong basic sites above 700 °C increases as the amount of triazole increases. PWT3 and PWT4 show a decrease in peak intensity around 550 and 600 °C, while a peak at 700 °C arises at the same time. Additionally, for PWT5, a peak at the higher temperature of 800 °C is formed, indicating an even stronger basic site. Thus, an increase in the triazole content of the PWA possibly facilitated the formation of strong basic sites, which is an important factor in the interaction with the reactants in a catalytic reaction. Therefore, we can conclude from the characterizations that the optimized triazole-PWA nanocomposites exhibited great structural stability, large surface areas, and bifunctional properties with moderate acidity and strong basicity, despite a conjugation of the PWA with triazole.

### 3.2. Transesterification of Rapeseed Oil with Methanol

**Figure 6:**
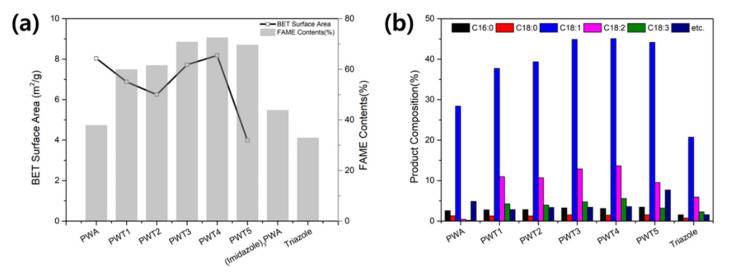
(**a**) FAME contents of the transesterification of rapeseed oil showing the BET surface areas and (**b**) the selected product compositions (C16:0, C18:0, C18:1, C18:2, C18:3).

As shown in Figure 6a, the transesterification of rapeseed oil was analyzed with a fixed methanol/rapeseed oil molar ratio of 10: 1 and 4 wt% of the catalyst (based on rapeseed oil) at 200 °C for 2 h. PWTx exhibited better catalytic performance than the pristine PWA and triazole, which showed only 37.79% and 32.88% conversion, respectively. Furthermore, on reacting the same heterocycles with imidazole, the yield (43.78%) was approximately 20% lower than that obtained using the triazole composite (61.5%). Thus, the enhanced catalytic performance of PWTx could be attributed to bifunctional catalysis by its basic and acidic sites (provided by triazole and the PWA, respectively) [40,41]. Additionally, the triazole-PWA composites exhibited different catalytic activities according to their composition; as the triazole content increased, the reactivity increased, along with the combination of relatively high surface area and appropriate bifunctionality with strong basic sites. However, although PWT5 had the smallest particle size and the proper bifunctional composition, it exhibited a low catalytic activity due to its low surface area from the distortion of the pore structure by incorporating excess triazole into the PWA.

As shown in Figure 6b, the composition ratio of the biodiesel products (such as methyl palmitate (C16:0), methyl stearate (C18:0), methyl oleate (C18:1), methyl linoleate (C18:2), and methyl linolenate (C18:3)) was analyzed. The formation of methyl oleate (C18:1) showed the same tendency as that exhibited by the sum of all the biodiesel components (shown in Figure 6a). However, the methyl linoleic acid (C18:2) content decreased steeply using the PWA catalyst (0.47%). The rapid decrease in the amount of C18:2 could be correlated with the increase in C18:1 due to the trade-off effect with a strong acid capacity [42]. Rapeseed oil is composed of 90% unsaturated fatty acids (methyl oleate (C18:1), methyl linoleate (C18:2), and methyl linolenate (C18:3)). Therefore, a highly acidic catalyst with sufficient basic sites should efficiently convert the unsaturated fatty acids in rapeseed oil to FAME. Thus, the synergy of bifunctional triazole/PWA showed a high conversion efficiency of rapeseed oil to FAME.

## 4. Conclusions

A PWTx bifunctional nanocomposite was designed using the precipitation method to use during the transesterification of rapeseed oil to produce biodiesel. The synthesized materials were physically characterized by XRD and SEM, which indicated a composite structure and regularly shaped nanoparticles with different sizes, respectively. The chemical characterization (using FT-IR and XPS) of PWTx confirmed the structural changes of the heteropoly acid caused by triazole addition. Furthermore, the triazole-functionalized PWA nanoparticles exhibited significantly higher thermal stability than the pristine PWA nanoparticles. Their basic and acidic properties were analyzed using NH_3_-TPD and CO_2_-TPD, respectively. The total acidic sites in PWTx did not exhibit a significant change, while the increasing triazole content facilitated the number of strong basic sites. The enhanced catalytic performance of PWTx could be attributed to bifunctional catalysis by its acidic and enhanced basic sites. Notably, the catalytic performance of PWTx exhibited a volcano-shaped plot with respect to its triazole content. Therefore, we found that the PWA-composite nanoparticles of PWT4 have an optimized bifunctional structure and high surface area, resulting in high FAME-conversion performance, which shows the possibility of replacing the commercial catalysts with great advantages. In addition, depending on the reaction conditions and mechanism of the catalytic reaction, the possibility of developing nanocomposites utilizing various types of heteropoly acids is infinite.

## Figures and Tables

**Table 1:** Element analysis (EA) results of the synthesized catalysts and pH changes of the synthesis solution when increasing the number of triazole moieties.

	Triazole/PWA Molar Ratio	Carbon (wt%)	Nitrogen (wt%)	pH
PWT1	1	2.36	4.04	1.8
PWT2	3	2.41	3.98	3.4
PWT3	4	2.40	4.06	4.7
PWT4	6	2.44	4.06	4.9
PWT5	10	2.38	4.08	5.0

**Table 2:** Physical properties of PWA and the synthesized catalysts.

	Surface Area (m^2^/g)	Pore Volume (cm^3^/g)	Ave. Pore Diameter (nm)
PWA	8.0337	1.8458	34.657
PWT1	6.8811	1.581	45.511
PWT2	6.2458	1.435	48.98
PWT3	7.7215	1.774	36.201
PWT4	8.181	1.8796	40.952
PWT5	3.9909	0.9169	66.4

**Table 3:** Acidity and basicity capacities calculated using NH_3_-TPD and CO_2_-TPD, respectively, for PWTx.

	Acidity Capacity (mmol/g)	Basicity Capacity (mmol/g)
PWT1	28.27	17.33
PWT2	28.69	20.53
PWT3	29.41	13.37
PWT4	29.07	11.61
PWT5	28.02	10.62

## Data Availability

The data presented in this study are available on request from the corresponding author.
